# Tantalum versus titanium acetabular component in single-stage hip revision for periprosthetic joint infection: a comparative analysis of implant survivorship

**DOI:** 10.1186/s10195-025-00867-6

**Published:** 2025-07-23

**Authors:** Zhaoxi Xue, Wentao Guo, Wenbo Mu, Boyong Xu, Li Cao

**Affiliations:** 1 Department of Orthopaedics, The First Affiliated Hospital of Xinjiang Medical University, 137 South LiYuShan Road, 830054 Urumqi, Xinjiang China; 2 Ministry of Education, Key Laboratory of High Incidence Disease Research in Xinjiang (Xinjiang Medical University, Ürümqi, Xinjiang China; 3 Xinjiang Clinical Research Center for Orthopedics, Ürümqi, Xinjiang China

**Keywords:** Tantalum, Acetabular reconstruction, Single-stage revision

## Abstract

**Background:**

The impact of tantalum (Ta) versus titanium (Ti) acetabular components on reinfection risk in periprosthetic joint infection (PJI) remains controversial. While prior studies have focused on two-stage revisions, this is the first comparative analysis of Ta versus Ti in single-stage revisions. This study aimed to compare all-cause rerevision and infection recurrence rates between Ta and Ti acetabular components in single-stage revision for chronic PJI.

**Materials and methods:**

In this study, all patients underwent single-stage revision combined with intra-articular (IA) antibiotic infusion, with 56 receiving Ta acetabular components and 79 receiving Ti components. Both the Ta and Ti groups utilized acetabular reconstruction methods (including cups with and without augments) and cementless prostheses for all femoral components. We compared implant survivorship between the two groups, using implant survivorship free from reinfection and all-cause revision as the endpoints. Multivariate logistic regression (MVLR) was used to determine the independent predictive factors for septic failure.

**Results:**

The implant survivorship free from reinfection of the Ta group (92.9%; 95% confidence interval (CI) 85.7~98.2%) was comparable to that of the Ti group (88.6%; 95% CI 81.0~94.9%; *P* = 0.391; log-rank test). The implant survivorship free from all-cause rerevision of the Ta group (91.1%; 95% CI 84.1~100%) was comparable to that of the Ti group (87.3%; 95% CI 78.9~94.4%; *P* = 0.323; log-rank test). MVLR did not identify the Ta acetabular component (*P* = 0.414) as a protective factor against septic failure in acetabular reconstruction. However, previous revision (*P* = 0.048) was identified as a risk factor.

**Conclusions:**

Ta acetabular components exhibited a risk of all-cause rerevision comparable to Ti components in single-stage revision, with no significant protective effect against reinfection. These findings suggest that the notion of Ta components preventing infections should be viewed with caution.

## Introduction

Periprosthetic joint infection (PJI) is one of the most devastating complications following total hip arthroplasty (THA). Acetabular revision using uncemented components is widely practiced [1]. Most of these components are made from tantalum (Ta) or titanium (Ti) [2]. Ta is favored for its higher porosity, superior friction coefficient, and excellent elastic modulus [3]. A single-center study of 144 THAs revised specifically for PJI observed that using Ta acetabular component in revision THA for septic failure can reduce the risk of subsequent reinfection [4]. Other randomized studies using national registries found no survival benefit in terms of revision for infection attributable to Ta acetabular component [5, 6]. These studies focused on revision cases in the two-stage revision, and no study has compared the effects of Ta and Ti on reinfection rates in single-stage revision.

The primary reason for single-stage revision underutilization lies in strict patient selection criteria. In most regions, single-stage revision was only cautiously used in patients without sinus tracts, with a definite infective microorganism and effective antibiotics available, and in patients who had no history of previous surgeries. Currently, the Endo-Klinik in Hamburg considers only culture-negative PJI and preoperative sepsis as absolute contraindications for single-stage revision. However, studies by van den Kieboom from Harvard Medical School and Carsten Perka from the Charité University Hospital in Berlin have shown that preoperative culture-negative PJI did not affect the infection control rate in single-stage revision [7–10]. Previous studies have demonstrated that combining single-stage revision with intra-articular (IA) antibiotic infusion can achieve favorable outcomes in patients with sinus tracts, polymicrobial infections, culture-negative PJI, and multiple failed surgeries [11–14].

This comparative study evaluated all-cause rerevision and infection recurrence rates following single-stage acetabular reconstruction in PJI cases, comparing Ta and Ti acetabular components, both used with intra-articular (IA) antibiotic infusion. Our primary objective was to determine whether Ta acetabular components provide superior protection against recurrent infections compared with Ti components in single-stage revision following septic failure. Secondly, we aimed to identify risk factors associated with postoperative septic failure.

## Materials and methods

**Fig. 1:**
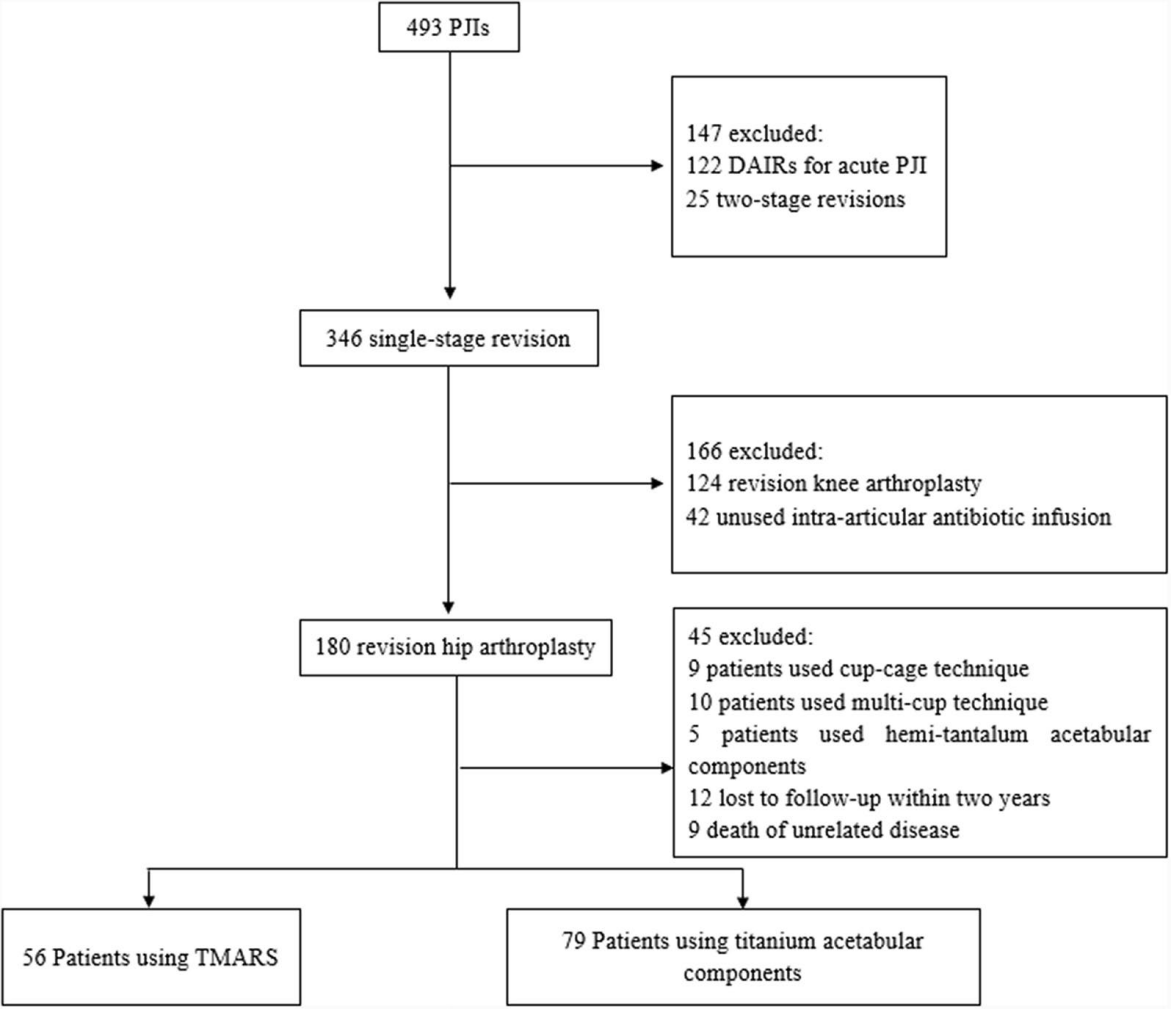
Flowchart showing the constitution of the cohort

Institutional review board approval was secured for this study. We conducted a retrospective analysis of our electronic database from January 2013 to February 2023 to identify patients who received cementless Ta and Ti acetabular components during single-stage revision for PJI. The study flow diagram is shown in Fig. 1.

Inclusion criteria were the following: (i) patients with periprosthetic joint infection; (ii) patients who received cementless Ta and Ti acetabular components, with or without augments; and (iii) patients with adequate follow-up and complete data.

**Fig. 2:**
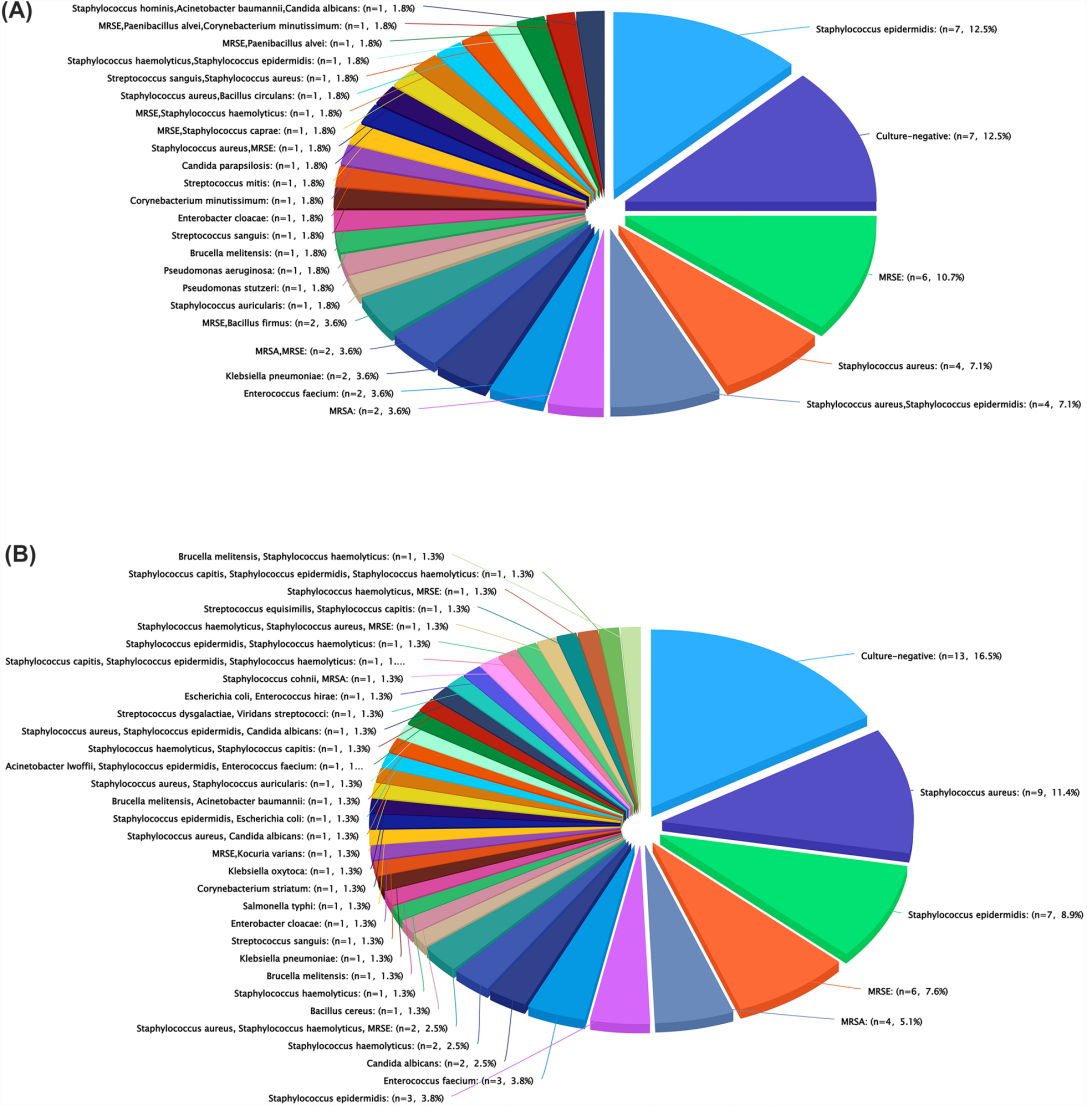
**A** The composition of microorganisms in the tantalum group. **B** The composition of microorganisms in the titanium group

Exclusion criteria were the following: (i) non-PJI patients; (ii) patients who did not undergo revision of the acetabular component; (iii) patients who had a revision with cemented acetabular components; (iv) patients who underwent revision using “cup-cage,” “cup-in-cup,” or “cup-on-cup” techniques; and (v) patients without adequate follow-up and complete data. The types and manufacturers of acetabular cups used in both Ta and Ti groups, along with their respective liner fixation methods, are detailed in Table 1. All patients diagnosed with PJI met the Musculoskeletal Infection Society’s criteria [15]. PJI was diagnosed according to the Musculoskeletal Infection Society (MSIS) criteria in patients who either met one major criterion or fulfilled more than three minor criteria. The microorganisms of each patient are listed in Fig. 2. “Culture-negative” was interpreted as no growth of any pathogen in aerobic and anaerobic cultures taken from the preoperative joint aspiration and the intraoperative periprosthetic tissue samples. For patients with negative cultures, PJI was established through other diagnostic criteria, excluding those based on culture results. The exclusion criteria for our single-stage procedure are presented in Table 2. There were 56 cases of patients treated with Ta acetabular components and 79 cases involving the use of Ti acetabular components. Age, gender, body mass index (BMI), hypertension, diabetes, C-reactive protein (CRP), erythrocyte sedimentation rate (ESR), acetabular reconstruction methods, acetabular bone defects, sinus tract, stem revised, polymicrobial infection, culture-negative PJI, extended trochanteric osteotomy (ETO), bone graft (acetabular), bone graft (femoral), bearing surface, and primary revision were strictly recorded (Table 3). The Paprosky classification system was used to classify all the acetabular deficiencies [16]. Two authors reviewed the preoperative radiographs and confirmed the acetabular deficiency classifications through intraoperative verification.

**Table 1:** Porous tantalum and the other commonly used uncemented cups

Group	Cup design	Liner fixation methods	Case, *n* (%)
Ta	TM shell (Zimmer Biomet)	Cemented fixation	56 (41.5)
Ti	R3 (Smith & Nephew)	Cemented fixation	19 (14.1)
	R3 (Smith & Nephew)	Locking mechanism	39 (28.9)
	Pinnacle (DePuy Synthes)	Locking mechanism	11 (8.1)
	Porocoat (DePuy Synthes)	Locking mechanism	5 (3.7)
	We-Done I (Star Joint)	Locking mechanism	5 (3.7)

**Table 2:** Exclusion criteria of single-stage revision

Exclusion criteria
1. Severely immunocompromised patients categorized according to the Musculoskeletal Infection Society (MSIS) staging system 2. Patients with active systemic infections such as septicemia 3. Patients with severe coexisting medical conditions who are unable to tolerate surgery 4. The periarticular soft tissue is extensively damaged, and joint function cannot be restored through revision surgery 5. The infection has spread widely to other cavities, making thorough eradication impossible 6. The infection involves the neurovascular bundles and peripheral vascular disease

**Table 3:** Comparison of demographic factors between Ta group and Ti group

Patients	Ta (*n* = 56)	Ti (*n* = 79)	*P*-value
Mean age, IQR	65(51,71)	60(48,72)	0.352
Sex			
Men, *n*	27	46	0.250
Women, *n*	29	33	
Mean body mass index, IQR	25(23,27)	25(20,27)	0.439
Hypertension			
Yes	15	16	0.374
No	41	63	
Diabetes			
Yes	6	8	0.912
No	50	71	
CRP	17(6,36)	18(11,46)	0.399
ESR	43(24,63)	44(24,60)	0.704
Acetabular bone defects			
I	9	21	0.091
IIA	17	21	
IIB	9	20	
IIC	12	10	
IIIA	9	7	
Sinus tract			
Yes	16	27	0.491
No	40	52	
Stem revised			
Yes	49	71	0.666
No	7	8	
Polymicrobial infection			
Yes	18	21	0.482
No	38	58	
Culture-negative PJI			
Yes	7	13	0.524
No	49	66	
Acetabular reconstruction methods			
Cup with augment	12	8	0.069
Cup without augment	44	71	
Bone graft (acetabular)			
Yes	7	8	0.666
No	49	71	
Bone graft (femoral)			
Yes	3	4	0.616
No	53	75	
ETO			
Yes	8	22	0.062
No	48	57	
Bearing surface			
MOP	13	11	0.164
COP	43	68	
Primary revision			
Yes	20	17	0.068
No	36	62	

*CRP* C-reactive protein, *ESR* erythrocyte sedimentation rate, *ETO* extended trochanteric osteotomy, *IQR* interquartile range, *MoP* metal-on-polyethylene, *CoP* ceramic-on-polyethylene

### Surgical methods

All surgical procedures were performed by the same surgeon, using a posterior lateral approach to the hip. After joint capsule incision, a minimum of three tissue samples were collected for culture, sensitivity tests, and histological evaluation. Following radical debridement, the surgical area was exhaustively irrigated. It was irrigated with 3–10 L of saline and 100–200 mL of 3% hydrogen peroxide. Then, all soft tissues were soaked for 15 min in 400–500 mL of 0.5% aqueous betadine. The acetabulum was prepared using suitable files until fresh bleeding or exposed trabeculae were evident in the host bone bed.

The prosthetic and augment trial molds were inserted according to the acetabular deficiency morphology, with additional filing of the acetabular bone surface if necessary for a secure fit and initial stability. The cup and augment were clamped and inserted into the acetabulum on the basis of trial placement and angle, and they were impacted until achieving firm initial stability and a tight bond with the bone surface. If they were not firm enough, bone cement was used to bond the cup and augment before hitting them together. The cup and augment were screwed into the appropriate angle and number of screws for fixing. In cases where the cup position was suboptimal or the number of screw holes insufficient, high-speed drills were employed to create additional holes for fixing cup and augment. Unused screw holes were sealed with bone wax to prevent cement penetration into the cup-host bone interface. In the Ta group, the liner’s angle was adjusted using 0.5 g of gentamicin-loaded commercial bone cement (Zimmer Biomet Orthopaedics) to achieve the desired abduction and anteversion angles. In the Ti group, 24% (19/79) of the liners were fixed with cemented fixation, while the remaining liners were secured using locking mechanism (Table 1). On the femoral side, we routinely used a cementless prosthesis, typically a double-tapered rectangular stem or a tapered multipoint-fixed prosthesis. Femora undergoing ETO were stabilized with titanium cerclage bands or wire cables. 

A three-branch catheter was inserted into the intra-articular space of the hip for postoperative antibiotic delivery, a suction drain was placed at the distal hip, and 0.5 g of vancomycin (VAN) or meropenem (MEM) were placed deep into the fascia and closed in a standard fashion. All individual antibiotic regimens were selected collaboratively by the treating surgeon and the local clinical microbiology department. The initial antibiotic regimen was guided by preoperative joint fluid specimens and was later adjusted according to intraoperative cultures. The postoperative antibiotic regimen is presented in Table 4 [14].

**Table 4:** Regimens of antibiotic application for patients

Microorganism	Antibiotics	Medication regimen	
		Approach	Dosage
Gram-positive bacteria	VAN	IA + IV	IV: 1 g, q12h; IA: 0.5 g, qd
Gram-negative bacteria	MEM	IA + IV	IV:1 g, q8h; IA: 0.5 g, qd
Gram-positive bacteria plus gram-negative bacteria	VAN MEM	IA + IV	IV: 1 g, q12h; IA: 0.5 g, qd IV: 1 g, q8h; IA: 0.5 g, qd
Gram-positive bacteria plus fungus	VAN	IA + IV	IV: 1 g, q12h; IA: 0.5 g, qd
	Fluconazole		IV: 0.2 g, q12h; IA: 0.2 g, qd
Culture-negative	VAN MEM	IA + IV	IV: 1 g, q12h; IA: 0.5 g, qd IA: 0.5 g, qd

*IV* intravenous, *IA* intra-articular, *VAN* vancomycin, *MEM* meropenem, *q12h* every 12 hours, *q8h* every 8 hours, *qd* once a day

For all patients, intravenous (IV) antibiotics were administered for an average of 11 days (range 7–20 days) and IA antibiotics for an average of 14 days (range 12–28 days). Among these patients, those with polymicrobial infections received IV antibiotics for an average of 13 days (range 12–18 days) and IA antibiotics for an average of 15 days (range 13–28 days). In addition, patients with culture-negative PJI had IV antibiotics for an average of 14 days (range 11–17 days) and IA antibiotics for an average of 14 days (range 12–19 days). Patients with multiple failed surgeries received IV antibiotics for an average of 13 days (range 10–18 days) and IA antibiotics for an average of 16 days (range 12–21 days). IA administration was alternated to avoid drug interactions. Before IA antibiotics, synovial fluid was drawn for biochemical testing after closing the drainage tube, and the tube was reopened 6 h after IA administration for 18 h before the next injection. The discontinued criteria were consistent with our previous study, including CRP 10 mg/L, synovial white blood cell count 1500 cells/µL, synovial fluid polymorphonuclear neutrophils 70%, and clear synovial fluid. Following hospital discharge, levofloxacin and rifampicin were jointly prescribed for oral switch therapy, and this therapy lasted 1–3 months. This therapy continued until C-reactive protein (CRP) and erythrocyte sedimentation rate (ESR) levels normalized or stabilized at near-normal limits. 

### Postoperative management

Weight-bearing timelines depended on prosthesis stability and bone defect severity. Patients with minor deficiency and stable prostheses commenced partial weight-bearing at 2 weeks, while those with significant deficiency started at 4 weeks and progressed to full weight-bearing at 8 weeks.

### Statistical analysis

SPSS 26.0 software was used for statistical analysis. The Kolmogorov–Smirnov test assessed data normality, with results expressed as mean ± standard deviation or median and interquartile range, as appropriate. Paired sample *t*-tests compared pre- and postoperative indices, while between-group comparisons utilized independent samples *t*-tests. The Wilcoxon test was used for two-group comparisons, respectively, considering *P* < 0.05 as statistically significant. The chi-squared test and Fisher’s exact test assessed differences in infection control rates among groups with varying infection profiles. Kaplan–Meier analysis was used to calculate infection-free survival rates. The log-rank test compared these rates between the Ta and Ti groups. Multivariate logistic regression (MVLR) was used to determine the independent predictive factors for septic failure.

## Results

### Demographic data

In the Ta group, 56 patients were followed up for an average of 5.0 years (ranging from 2.0 to 11.6 years), while in the Ti group, 79 patients were followed up for an average of 5.6 years (ranging from 2.0 to 11.3 years). The two groups were comparable in terms of acetabular reconstruction methods, acetabular bone defects, sinus tract, stem revision, polymicrobial infection, culture-negative PJI, and previous revision history (Table 3).

### Clinical outcomes

The complications of the Ta group included four patients (7.1%, 4/56) who developed recurrent infections, and their specific details are presented in Table 5. One patient (1.8%, 1/56) developed aseptic loosening and subsequently underwent revision surgery, with a favorable recovery outcome. There were two cases (3.6%, 2/56) of prosthetic dislocation, all successfully managed with closed reduction.

**Table 5:** The specific situation of patients with recurrent infections

Group	Number	Bone deficiency	Number of previous revisions	Operation types	Microorganism	Subsequent treatment
Ta	1	I	0	Single cup reconstruction	MRSA; MRSE	Debridement, antibiotics, and implant retention (DAIR)
Ta	2	IIC	0	Cup with augment	MRSE	DAIR
Ta	3	IIB	1	Single cup reconstruction	*Staphylococcus epidermidis*	Single-stage revision
Ta	4	IIA	1	Single cup reconstruction	MRSA	Single-stage revision
Ti	1	IIB	0	Single cup reconstruction	MRSE; *Kocuria varians*	Single-stage revision
Ti	2	IIB	3	Single cup reconstruction	*Staphylococcus aureus*; *Candida albicans*	Single-stage revision
Ti	3	I	0	Single cup reconstruction	*Brucella melitensis*	Single-stage revision
Ti	4	I	0	Single cup reconstruction	*Klebsiella pneumoniae*; *Escherichia coli*	Single-stage revision
Ti	5	IIA	1	Single cup reconstruction	*Staphylococcus aureus*; *Staphylococcus auricularis*	Single-stage revision
Ti	6	IIIA	0	Single cup reconstruction	Culture-negative	DAIR
Ti	7	IIA	0	Single cup reconstruction	Culture-negative	Single-stage revision
Ti	8	IIB	1	Single cup reconstruction	*Staphylococcus aureus*; *Staphylococcus epidermidis*; *Candida albicans*	Two-stage revision
Ti	9	I	1	Single cup reconstruction	*Staphylococcus capitis*; *Staphylococcus epidermidis*; *Staphylococcus haemolyticus*	DAIR

The complications of the Ti group included nine patients (11.3%, 9/79) who developed recurrent infections (Table 5). There were four cases (5.1%, 4/79) of prosthetic dislocation: one (1.3%, 1/79) was successfully managed with closed reduction, two (2.6%, 2/79) required open reductions, and one (1.3%, 1/79) underwent a one-stage revision.

### Reinfection in patients with Ta and Ti components

The infection recurrence rates of the Ta group (7.1%, 4/56) were comparable to that of the Ti group (11.4%, 9/79; *P* = 0.557; Fisher’s exact test). Excluding cases involving cups with augments and considering only those with cups without augments, the infection recurrence rates in the Ta group (6.8%, 3/44) were similar to those in the Ti group (12.6%, 9/71; *P* = 0.368; Fisher’s exact test).

### Survivorship from revision

**Fig. 3:**
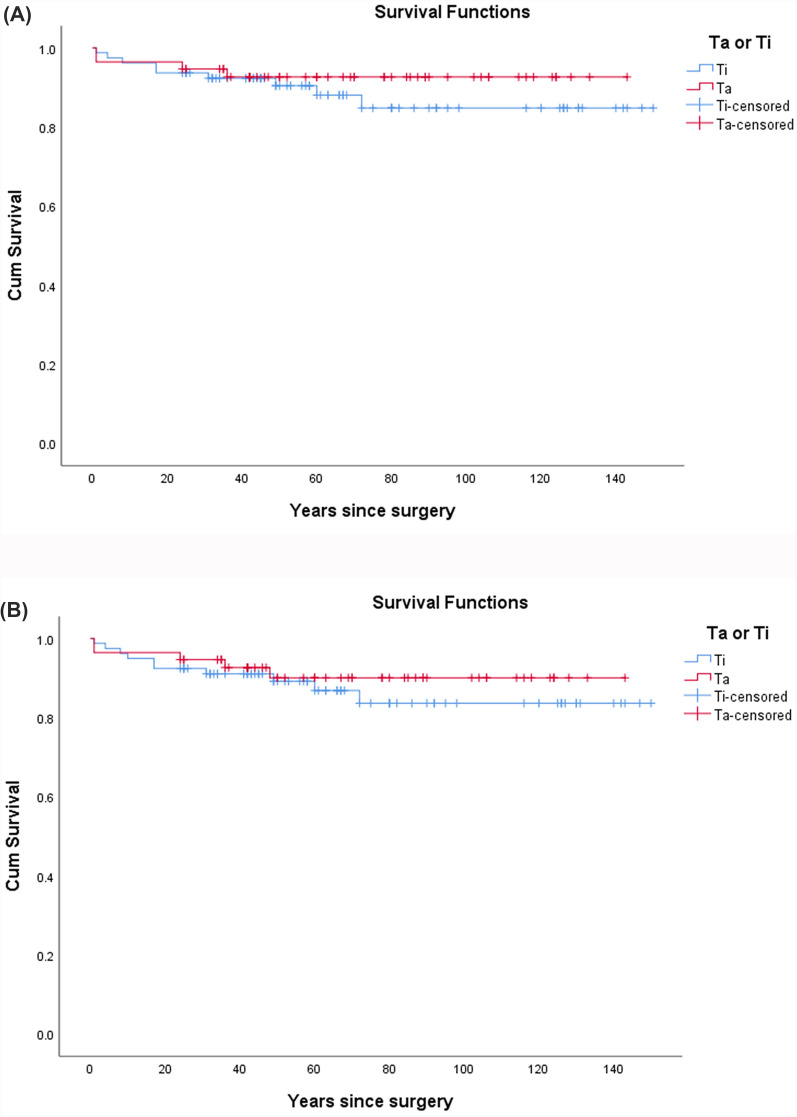
**A** The Kaplan–Meier survivorship free from rerevision for reinfection was compared between the tantalum group and the titanium group. **B** The Kaplan–Meier survivorship free from rerevision for all-cause was compared between the tantalum group and the titanium group

Using rerevision attributable to reinfection as the endpoint, the survival of the Ta group (92.9%; 95% CI 85.7~98.2%) was comparable to that of the Ti group (88.6%; 95% CI 81.0~94.9%; *P* = 0.391; log-rank test, Fig. 3A). Excluding cases involving cups with augments and considering only those with cups without augments, the survival of the Ta group (93.2%; 95% CI 84.1~100%) was comparable to that of the Ti group (87.3%; 95% CI 78.9~94.4%; *P* = 0.323). Using rerevision attributable to all-cause as the endpoint, the survival of the Ta group (91.1%; 95% CI 82.1~98.2%) was comparable to that of the Ti group (87.3%; 95% CI 79.7~93.7%; *P* = 0.478; log-rank test, Fig. 3B). Excluding cases involving cups with augments and considering only those with cups without augments, the survival of the Ta group (90.9%; 95% CI 81.8~97.7%) was comparable to that of the Ti group (87.3%; 95% CI 78.9%–94.4%; *P* = 0.567).

### Univariable and multivariable logistic regression analysis of factors associated with reinfection

Univariable analysis found that polymicrobial infection (*P* = 0.045, odds ratio (OR) = 3.281, 95% CI 1.018~10.903) and previous revision (*P* = 0.027, OR = 3.741, 95% CI 1.156~12.491) were positively associated with reinfection, whereas CRP (*P* = 0.049, OR = 0.993, 95% CI 0.986~1.001) was negatively associated with reinfection (Table 4). Multivariate analysis found that previous revision was (*P* = 0.048, OR = 3.372, 95% CI 1.002~11.686) positively associated with reinfection (Table 5). No association was found between the type of acetabular components and reinfection (*P* = 0.414) Tables 6, 7

**Table 6:** Univariate logistic regression for reinfection

Patients	Failed	Did not fail	*P*-value	OR	95% CI
Mean age, years; IQR	62 (44, 70)	61 (50, 72)	0.216	1.024	0.986~1.063
Sex					
Men, *n*	8	65	0.571	1.403	0.443~4.869
Women, *n*	5	57			
Mean body mass index, IQR	26 (21, 29)	25 (21, 26)	0.452	0.943	0.809~1.100
CRP	31 (6, 85)	17 (8, 42)	**0.049**	0.993	0.986~1.001
ESR	50 (18, 64)	42 (25, 60)	0.123	0.988	0.970~1.004
Hypertension					
Yes	3	28	0.992	1.007	0.215~3.562
No	10	94			
Diabetes					
Yes	2	12	0.537	1.667	0.240~7.219
No	11	110			
Type of acetabular components					
Ta	4	52	0.414	0.598	0.155~1.946
Ti	9	70			
Acetabular bone defects					
IIA	4	34	0.469	1.839	0.403~14.186
IIB	4	25	0.738	1.292	0.309~7.748
IIC	1	21	0.617	0.663	0.090~3.080
IIIA	1	15	0.400	0.561	0.114~2.045
Sinus tract					
Yes	6	37	0.251	1.969	0.597~6.324
No	7	85			
Stem revised					
Yes	12	108	0.682	1.556	0.273~29.403
No	1	14			
Polymicrobial infection					
Yes	7	32	**0.045**	3.281	1.018~10.903
No	6	90			
Culture-negative PJI					
Yes	2	18	0.952	1.051	0.154~4.348
No	11	104			
Acetabular reconstruction methods					
Cup with augment	1	19	0.458	0.452	0.024~2.506
Cup without augment	12	103			
ETO					
Yes	4	26	0.439	1.641	0.418~5.486
No	9	96			
Bone graft (acetabular)					
Yes	2	13	0.608	1.524	0.220~6.534
No	11	109			
Bone graft (femoral)					
Yes	1	3	0.317	3.306	0.157~28.23
No	12	119			
Bearing surface					
MOP	3	21	0.601	0.693	0.192~3.284
COP	10	101			
Previous revision					
Yes	7	29	**0.027**	3.741	1.156~12.491
No	6	93			

Bold refers to statistical significance (*P* < 0.05);*ETO* extended trochanteric osteotomy, *MoP* metal-on-polyethylene, *CoP* ceramic-on-polyethylene, *OR* odds ratio

**Table 7:** Multivariate logistic regression for reinfection

Patients	Failed	Did not fail	*P*-value	OR	95% CI
CRP	31 (6, 85)	17 (8, 42)	0.076	0.993	0.987~1.001
Polymicrobial infection					
Yes	7	32	0.080	2.946	0.877~10.246
No	6	90			
Previous revision					
Yes	7	29	**0.048**	3.372	1.002~11.686
No	6	93			

Bold refers to statistical significance (*P* < 0.05). *OR* odds ratio

## Discussion

The difference between Ta and Ti components in septic revision remained controversial. Previous research has shown no difference in rerevision rates for PJI when Ta components were used for all-cause revision THAs. Ta components may reduce the risk of infection only when used specifically for PJI revisions, rather than for all-cause revision THAs [6]. However, research specifically focusing on the use of Ta components in PJI revisions is limited. This study was the first to compare the infection control rates of Ta and Ti acetabular components in single-stage revision for PJI. Although we did not observe a protective effect of Ta components in preventing revisions due to PJI, we identified previous revision surgery to be a risk factor.

A single-center study involving 144 THAs revised for PJI found that the use of Ta components was associated with a reduced incidence of subsequent infection in patients with PJI [4]. The authors proposed that Ta components may reduce infection risk through: (i) enhanced osseointegration, minimizing dead space for microbial colonization; (ii) lower bacterial adhesion owing to TM’s three-dimensional structure; and (iii) promotion of leukocyte activation, boosting local host defenses. In this regard, Harrison et al. showed no significant difference in antimicrobial or anti-biofilm properties between Ta and Ti in vitro experiments. They emphasized that bacterial colonization and biofilm formation can occur within hours, whereas osteoblastic adherence and subsequent bone ingrowth, along with the elimination of dead space, may take days to weeks. In addition, they noted that in the Ta group, antibiotic-loaded cement (polymethylmethacrylate) was used to secure the polyethylene liner, whereas in the Ti group, the polyethylene liner is secured via a rim locking mechanism. The use of antibiotic-loaded cement in the Ta group may contribute to the higher infection control rates observed [17].

In our study, the bone cement used was not high-dose antibiotic-loaded but rather a commercial gentamicin-loaded bone cement, containing 0.4 g of gentamicin per 40 g of cement powder. A previous study demonstrated that IA antibiotic infusion can help to achieve effective drug concentration in the serum and synovial fluid of patients with PJI [18, 19]. In this study, both the Ta and Ti groups employed IA administration to achieve significantly higher antibiotic concentrations within the joint cavity and to sustain them for longer periods, theoretically surpassing those attainable with antibiotic-loaded bone cement. Therefore, the potential influence of antibiotic-loaded cement as a confounding factor can be discounted. Despite this, we did not observe a reduction in infection risk associated with Ta components. There were numerical differences in implant survivorship free from infection (Ta: 92.9% (52/56) versus Ti: 88.6% (70/79)) and in implant survivorship free from all-cause rerevision (Ta: 91.1% (51/56) versus Ti: 87.3% (69/79)). However, these differences were not statistically significant. The small sample size may have contributed to this outcome, and it remains uncertain whether a larger sample would reveal a significant disparity. This finding aligns with a study from the National Joint Registry (NJR) for England and Wales, which also demonstrated that, in revision THAs performed for PJI, the risk of rerevision due to infection did not significantly differ between patients receiving Ta and Ti acetabular components [6].

We typically employed Ti acetabular components for patients exhibiting minor bone deficiency, whereas Ta acetabular components were used for those with major deficiency. Previous single-center studies, as well as many joint registries and large database studies, did not record treated acetabular defects [4–6, 20]. This raises the possibility that Ta components, which are more likely to be used in complex revisions involving larger acetabular defects, may have an increased risk of future rerevisions compared with Ti components [21]. To mitigate this potential bias, we excluded more severe cases (based on the use of “cup-cage,” “cup-in-cup,” or “cup-on-cup”) and performed a separate subgroup analysis excluding cases where augments were used. The subgroup analysis did not change our findings. Despite no significant difference in bone defects between the Ta and Ti groups (*P* = 0.091), logistic regression analysis revealed that Ta acetabular component (*P* = 0.414) was not a protective factor against acetabular component infection failure. However, previous revision (*P* = 0.048) was identified as a risk factor, which is consistent with previous studies.

One of the key strengths of this study lies in the comprehensive collection of data, which included detailed acetabular defect assessments along with microbiologic analyses of tissue and fluid samples obtained during revision surgery. These data are not routinely captured by registry databases. After sample analysis, some cases might not actually have had PJI. Similarly, the rerevision rates for subsequent infections could be somewhat different if these analyses had been taken into account [6]. In addition, registries do not collect data on non-revision procedures, including closed reductions or debridement, antibiotics, and implant retention (DAIR), which may lead to an underestimation of the incidence of postoperative complications [22]. This study included patients with sinus tracts, polymicrobial infections, and culture-negative PJI, improving the generalizability of the study [23]. These conditions are traditionally considered contraindications for single-stage revision. We found that single-stage revision combined with IA antibiotic infusion was effective for patients under the abovementioned conditions. Ji and Li et al. also showed that this approach can achieve infection control rates ranging from 89.2% to 94.3% [13, 14, 24]. These outcomes were consistent with those previously reported in studies focusing on both single-stage and two-stage revision [25, 26].

This study has several limitations. First, this study was conducted at a single center and reflected the outcomes of a single surgeon. The patient populations were relatively small, which limited the available data for analysis. Second, the revision cases for PJI were heterogeneous, involving varying pathogens, varying degrees of bone defects, and varying numbers of previous revisions, all of which could affect the results. These covariates introduce the potential for uncontrolled selection biases among subgroups, thereby impacting the statistical results. Third, the Ti group included a heterogeneous mix of uncemented cups. Despite this heterogeneity, all cups (both Ta and Ti) featured a highly porous coating. The investigation specifically focused on evaluating whether the material composition (Ta versus Ti) of the revision components impacted clinical outcomes. Finally, the observations from this study are limited to the specific acetabular components used and should not be extended to highly porous designs manufactured by other companies.

## Conclusions

In this study, Ta acetabular components demonstrated a risk of all-cause rerevision comparable to that of Ti components in single-stage revision. Moreover, we did not observe a protective effect of Ta acetabular components in preventing revisions due to reinfection. These results contrast with those of previous single-center studies, suggesting that the claim of Ta components preventing infections should be approached with caution. Further studies with larger sample sizes and longer follow-up durations are necessary to validate these findings and to explore whether differences in infection control rates between Ta and Ti components can be established.

## Data Availability

The data that support the findings of this study are available from the corresponding author upon reasonable request.
